# Controversies and outlooks on Vestibular Rehabilitation

**DOI:** 10.1590/S1808-86942011000200001

**Published:** 2015-10-19

**Authors:** Marco Aurélio Bottino, Maurício Malavasi Ganança, Roseli Saraiva Moreira Bittar, Mario Edvin Greters, Fernando Freitas Ganança

Vestibular rehabilitation (VR) is well accepted today as an efficacious treatment option for body balance disorders. In order to enjoy its full efficacy, it must be employed within criteria based on the patient's diagnosis and clinical situation. The Department of Neurotology of the Brazilian Association of Otorhinolaryngology and Neck and Facial Surgery has been receiving numerous questions about the current status of VR. The questions are basically associated with treatment: who is responsible for indicating it; who does it; which is the best method for a given disorder, etc.

In our view, it is only the patient's physician who can consider and indicate the best treatment option for his/her patient – VR, in this case. The medical professional who is following the patient must master the physiology of posture and movement and, also, how the central nervous system coordinates body balance when facing the tasks presented to the individual. There is no specific field in charge of VR.

Today, different fields of medicine and paramedicine have been dedicating studies to body balance. Among the specialists who work with this topic, the otorhinolaryngologist plays a fundamental role, because he/she is the professional who has the anatomo-physiological knowledge concerning the system responsible for movement perception and integration – the vestibular system. The ENT is also the professional who has the necessary skills to surgically approach the temporal bone.

Given the growing interest of our members, in 2011 our Department of Neurotology intends to host a symposium with the goal of presenting and discussing current views on VR, considering all its aspects. The “Controversies and Outlooks of Treatment in Neurotology” is scheduled for November of the present year, in a venue yet to be defined, in the city of São Paulo.

